# Distribution and antimicrobial resistance analysis of gram-negative bacilli isolated from a tertiary hospital in Central China: a 10-year retrospective study from 2012 to 2021

**DOI:** 10.3389/fmicb.2023.1297528

**Published:** 2023-12-04

**Authors:** Ting Shi, Liangyi Xie

**Affiliations:** Department of Clinical Laboratory, Hunan Provincial People’s Hospital (The First Affiliated Hospital of Hunan Normal University), Changsha, Hunan, China

**Keywords:** gram-negative bacilli, antimicrobial resistance, carbapenems, fluoroquinolones, Central China

## Abstract

**Background:**

Gram-negative bacilli are one of the most common causes of various infections in clinical. The emergence and global spread of multi-drug resistant gram-negative bacilli has become a major challenge in the global public health field.

**Methods:**

A total of 51,189 non-repetitive strains of gram-negative bacilli were isolated in clinical settings. The antimicrobial susceptibility testing was conducted by using the automated VITEK 2 compact system and the matched AST susceptibility test card, complemented by the disk diffusion method. The antimicrobial susceptibility results were interpreted by CLSI. Rates of MDR and XDR in *Klebsiella pneumoniae*, *Acinetobacter baumannii*, and *Pseudomonas aeruginosa* were investigated. Used the chi-square test to determine whether the antimicrobial resistance rates of four major gram-negative bacilli isolated from ICU and non-ICU department have statistical differences.

**Results:**

*Escherichia coli* (31.4%), *Klebsiella* spp. (21.2%), *Acinetobacter* spp. (13.8%), and *P. aeruginosa* (11.0%) were the most frequently isolated gram-negative bacilli. *Escherichia coli* was the top one organism isolated from urinary tract (68.4%), bloodstream (39.9%), body fluid (33.2%), wound and pus (37%), except for respiratory tract (8.8%). Whereas *Acinetobacter baumannii* and *K. pneumoniae* were the major isolated organisms from respiratory tract. *Acinetobacter baumannii* showed high resistance to fluoroquinolones, β-lactam/β-lactamase inhibitor combinations class, ceftazidime, cefepime, imipenem, and meropenem, the resistance rates reached more than 70%. Ceftazidime showed a lower resistance rate to *E. coli* than ceftriaxone. For *E. coli*, fluoroquinolones showed a high resistance rate (ciprofloxacin 61.36% and levofloxacin 53.97%), whereas amikacin, carbapenems exhibited a lower resistance rate fluctuating at 2%. *Acinetobacter baumannii* and *K. pneumoniae* showed rapid increases in carbapenem resistance whereas *E. coli* had the lowest resistance rate and remain stable at 2%. *Acinetobacter baumannii* exhibited the highest rate of MDR and XDR, reaching 60–80 and 45–55%, respectively. Compared to non-ICU departments, the resistance rates of four major gram-negative bacilli in the ICU department were much higher and the differences were statistically significant (*p* < 0.05).

**Conclusion:**

Amikacin, carbapenems, and piperacillin/tazobactam exhibited relatively high sensitivity, whereas fluoroquinolones showed high resistance rate whether they can be the first-line antimicrobials for empirical treatment of UTI should take more consideration. The gram-negative bacilli in ICU were more resistance than that in non-ICU. These findings are helpful for clinicians using antimicrobials reasonably.

## Introduction

Antimicrobial resistance (AMR) has become a global health threat and this threat is particularly severe in China. As one of the largest consumers of antibiotics, China used 162,000 tons of antibiotics for healthcare and agriculture in 2013 (Zhang et al., 2015). The percentage of outpatient and inpatient prescriptions containing antibiotics in primary healthcare institutions was still high, reaching 52.9 and 77.5%, respectively (Wang et al., 2014). The overuse and inappropriate use of antibiotics in humans and animals was the main cause of increased AMR. WHO research suggested that AMR will cause a 1.4–1.6% annual decline in GDP (WHO, 2014). By 2050, AMR is expected to lead to 10 million deaths every year, with a loss of up to $100,000 billion (Department of Health and Social Care, 2016). Gram-negative bacilli (GNB) were accounted for about 70% of all isolated strains in China, which are one of the most common causes of respiratory tract infections, bloodstream infections, and urinary tract infections (Peleg and Hooper, 2010). Furthermore, GNB are also responsible for nosocomial infections, including 45–70% of ventilator-associated pneumonia and 20–30% of catheter-associated bloodstream infections (Barbier et al., 2013; Farrington and Allon, 2019). Multi-drug resistant GNBs have become a major challenge in the effective prevention and treatment of bacterial infections and have also brought a societal economic burden for patients in China ($77 billion in 2017; Zhen et al., 2021). It has been estimated that the patients with multi-drug resistant infection had higher hospitalization costs ($3,391), longer hospital stays (5.48 days), and higher mortality rates (1.5%; Zhen et al., 2021).

In response to this dire scenario, the world’s governments have created national action plans to control of bacterial resistance and the management of antibiotics. In 2015, the World Health Organization (WHO) issued a strategy—*the Global Action Plan for Antimicrobial Resistance* to combat antibiotic resistance (World Health Organization, 2017). Meanwhile, the WHO established the Global Antibacterial Drug Resistance Monitoring System (GLASS), aiming to standardize the surveillance of AMR through global cooperation. Antimicrobial resistance control has been put into the G20 summit’s consensus. China promulgated a series of documents and guidelines and carried out special activities to strengthen the management of antimicrobial application and controlled the development of drug-resistant pathogens. For example, the National Drug Administration issued a document in 2003 to restrict the activity of retail pharmacies selling antimicrobials without a prescription (National Medical Products Administration, 2003). Two nationwide antimicrobial resistant surveillance system have been established in 2005. From 2011 to 2013, the Ministry of Health of China (MOH) conducted a special rectification campaign to reduce the use of antibiotics in secondary and tertiary hospitals for 3 years (Ministry of Health, 2011, 2012, 2013). MOH classified the antibiotics into non-restricted, restricted, and special antibiotics. Only doctors with senior professional titles could prescribe all antibiotics, while junior doctors could only prescribe non-restrictive antibiotics (Ministry of Health, 2012). In 2016, the National Health and Family Planning Commission of the People’s Republic of China released the National Action Plan to contain bacterial resistance (National Health and Family Planning Commission et al., 2016).

Due to different countries and regions having their own resistant pattern of organisms, surveillance of local resistant patterns will assist in guiding the rational use of antimicrobial agents and conduce to control the antimicrobial resistance. In this surveillance study, a total of 51,189 non-repetitive strains of GNB were collected, the antimicrobial resistance profiles of GNB from 2012 to 2021 were assessed. Moreover, the antimicrobial resistance rates of four major GNB isolated from ICU and non-ICU department were compared. Rates of MDR and XDR of four major GNB were be investigated. Dynamic monitoring of the distribution and antimicrobial resistance trends of GNB are of great significance for the rational use of antimicrobial agents.

## Methods

### Hospital setting

Our study was conducted in Hunan Provincial people’s Hospital (The First-Affiliated Hospital of Hunan Normal University) located in Changsha city, which is a 4,000-bed tertiary comprehensive hospital founded in 1912. This hospital was comprised of 34 clinical departments and 15 medical technology departments. Changsha is the capital of Hunan province, which located in the central of China. It is on the Xiangjiang River 30 miles (50 km) south of Dongting Lake. The metro area population of this city in 2020 was 4,578,000.

### Strains collection

All gram-negative strains of this study were collected from January 1, 2012 to December 31, 2021 by a sentinel tertiary hospital (Hunan Provincial People’s Hospital), which was a part of China’s Antimicrobial Resistant Surveillance System. Inpatient and outpatient samples were considered for analysis. Repetitive isolated species of the same patient in the same specimen type were excluded from analysis, only the first isolate was acceptable. The specimen types include sputum, BALF, blood, urine, bile, ascites, secretion, and other clinical cultures. Inoculated the specimens onto Columbia blood agar and MAC agar plates and cultured them in a 37°C, 5% CO_2_ condition for 24 h to isolate gram-negative bacilli. Then, used MALDI-TOF MS (biomérieux, l’Etoile, France) or VITEK 2 compact system (biomérieux, l’Etoile, France) to identify the gram-negative bacilli.

### Antimicrobial susceptibility testing

The antimicrobial susceptibility testing was conducted by using the automated VITEK 2 compact system and the matched AST susceptibility test card, which can determine the minimum inhibitory concentrations (MICs) of antimicrobials. Moreover, disk diffusion method following by the criteria of Clinical and Laboratory Standards Institute (CLSI) was performed as supplementary method. The drug susceptibility results could be divided into sensitive, intermediate, and resistant according to the CLSI standard (Clinical and Laboratory Standard Institute, 2021). The resistance rate of antimicrobial was the percentage of the number of isolates that were resistant to certain antimicrobials to the total number of isolates detected. Antimicrobial agents tested in this study included penicillins (ampicillin), cephalosporins (ceftazidime, cefatriaxone, cefepime, cefuroxime, and cefoxitin), β-lactam/β-lactamase inhibitor combinations (piperacillin/tazobactam, cefoperazone/sulbactam), aminoglycosides (amikacin, tobramycin, and gentamicin), fluoroquinolones (ciprofloxacin and levofloxacin), carbapenems (imipenem, meropenem), folate pathway inhibitors (trimethoprim-sulfamethoxazole), monobactams (aztreonam). *E. coli* ATCC 25922, and *P. aeruginosa* ATCC 27853 were the quality control strains.

Based on Magiorakos et al. (2012) reported, an international expert proposal for interim standard definitions for acquired resistance was proposed. Multidrug-resistance (MDR) was defined as non-susceptible to at least one agent tested in three or more antibiotic classes, extensively drug-resistance (XDR) was defined as non-susceptible at least one agent tested in all but two or fewer antibiotic classes. Antibiotic classes for *K. pneumoniae*: aminoglycosides, anti-pseudomonal penicillins/β-lactamase inhibitors, carbapenems, cephalosporins, cephamycins, fluoroquinolones, folate pathway inhibitors, glycylcyclines, monobactams, penicillins, penicillins/β-lactamase inhibitors, polymyxins, and tetracyclines. Antibiotic classes for *A. baumannii*: aminoglycosides, antipseudomonal carbapenems, antipseudomonal fluoroquinolones, antipseudomonal penicillins/β-lactamase inhibitors, extended-spectrum cephalosporins, folate pathway inhibitors, penicillins/β-lactamase inhibitors, polymyxins, and glycylcyclines. Antibiotic classes for *P. aeruginosa*: aminoglycosides, anti-pseudomonas cephalosporins, anti-pseudomonal carbapenems, anti-pseudomonal fluoroquinolones, and anti-pseudomonal penicillins/β-lactamase inhibitors and polymyxins.

### Statistical analysis

WHONET (version 5.6) was used for statistical analysis of data. The comparison of antimicrobial resistance rates of four major gram-negative bacilli isolated from ICU and non-ICU department were performed using SPSS22.0 for chi-square test. Used Mantel–Haenszel test to analyze linear trend, with *p* < 0.05 as the difference being statistically significant.

## Results

### The distribution of gram-negative bacilli

**Figure 1:**
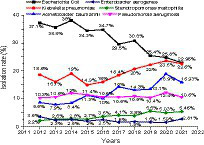
The isolation rates of the six major gram-negative bacilli between 2012 and 2021.

A total of 51,189 non-repetitive strains of gram-negative bacilli were isolated in clinical specimens from 2012 to 2021. The distribution of gram-negative bacilli in each year were shown in Table 1. The most frequently isolated gram-negative bacilli were *E. coli* (31.4%), *Klebsiella* spp. (21.2%), *Acinetobacter* spp. (13.8%), *P. aeruginosa* (11.0%), *Enterobacter* spp. (3.8%), and *S. maltophilia* (3.8%). These six species accounted for 85% of the total isolates. The 10-year isolation rates of the six major gram-negative bacilli were shown in Figure 1. We found that although *E. coli* was the highest isolation rate organism, the isolation rate showed a fluctuating downward trend in the past 10 years. Whereas the isolation rate of *K. pneumoniae* and *A. baumannii* showed an overall upward trend except declined in 2021. The isolation rate of *P. aeruginosa*, *S. maltophilia*, and *E. aerogenes* tends to be stable.

**Table 1:** Distribution of clinically isolated gram-negative bacilli between 2012 and 2021.

Type	2012	2013	2014	2015	2016	2017	2018	2019	2020	2021
*E. coli*	1,371 (37.1)	1,463 (35.6)	1,633 (38.1)	1,580 (34.3)	1,581 (34.8)	1,378 (29.6)	1,569 (30.8)	1715 (25.5)	1,588 (24.8)	1,611 (23.0)
*Klebsiella* spp.	800 (21.7)	819 (19.9)	886 (20.7)	852 (18.5)	818 (18.0)	939 (20.2)	1,112 (21.9)	1,541 (22.9)	1,554 (24.3)	1,656 (23.6)
*Acinetobacter* spp.	397 (10.7)	469 (11.4)	434 (10.1)	558 (12.1)	490 (10.8)	714 (15.3)	730 (14.3)	986 (14.7)	1,278 (19.9)	1,286 (18.3)
*P. aeruginosa*	383 (10.4)	440 (10.7)	519 (12.1)	527 (11.5)	486 (10.7)	496 (10.6)	559 (11.0)	701 (10.4)	788 (12.3)	744 (10.6)
*Enterobacter* spp.	318 (8.6)	287 (7.0)	147 (3.4)	109 (2.4)	132 (2.9)	100 (2.1)	118 (2.3)	149 (2.2)	167 (2.6)	333 (4.7)
*S. maltophilia*	82 (2.2)	111 (2.7)	122 (2.8)	125 (2.7)	168 (3.7)	193 (4.1)	201 (3.9)	376 (5.6)	322 (5.0)	383 (5.5)
*B. cepacia*	22 (0.6)	10 (0.2)	30 (0.7)	29 (0.6)	40 (0.9)	47 (1.0)	57 (1.1)	74 (1.1)	54 (0.8)	69 (1.0)
*H. influenzae*	-	-	60 (1.4)	356 (7.7)	367 (8.1)	279 (6.0)	258 (5.1)	449 (6.7)	62 (1.0)	338 (4.8)
*Proteus* spp.	67 (1.8)	63 (1.5)	87 (2.0)	44 (1.0)	40 (0.9)	62 (1.3)	75 (1.5)	62 (0.9)	62 (1.0)	89 (1.3)
*Serratia* spp.	61 (1.7)	100 (2.4)	59 (1.4)	77 (1.7)	96 (2.1)	65 (1.4)	45 (0.9)	82 (1.2)	78 (1,2)	95 (1.4)
*Citrobacter* spp.	53 (1.4)	49 (1.2)	47 (1.1)	38 (0.8)	50 (1.1)	45 (1.0)	54 (1.1)	60 (0.9)	43 (0.7)	70 (1.0)
*M. morganii*	16 (0.4)	15 (0.4)	6 (0.1)	1 (0.02)	4 (0.1)	2 (0.04)	8 (0.1)	13 (0.2)	11 (0.2)	45 (0.6)
*Aeromonas* spp.	3 (0.08)	8 (0.2)	12 (0.3)	9 (0.2)	12 (0.3)	8 (0.2)	7 (0.1)	16 (0.2)	28 (0.4)	46 (0.7)
Other	119 (3.3)	280 (6.8)	248 (5.8)	295 (6.5)	262 (5.6)	330 (7.2)	296 (5.9)	504 (7.5)	366 (5.8)	252 (3.5)

The number before the brackets represents the total number of bacteria isolated, and the data in the brackets represent the percentage. The symbol “-” represents the empty set.

### Distribution of organisms isolated in different specimens

**Figure 2:**
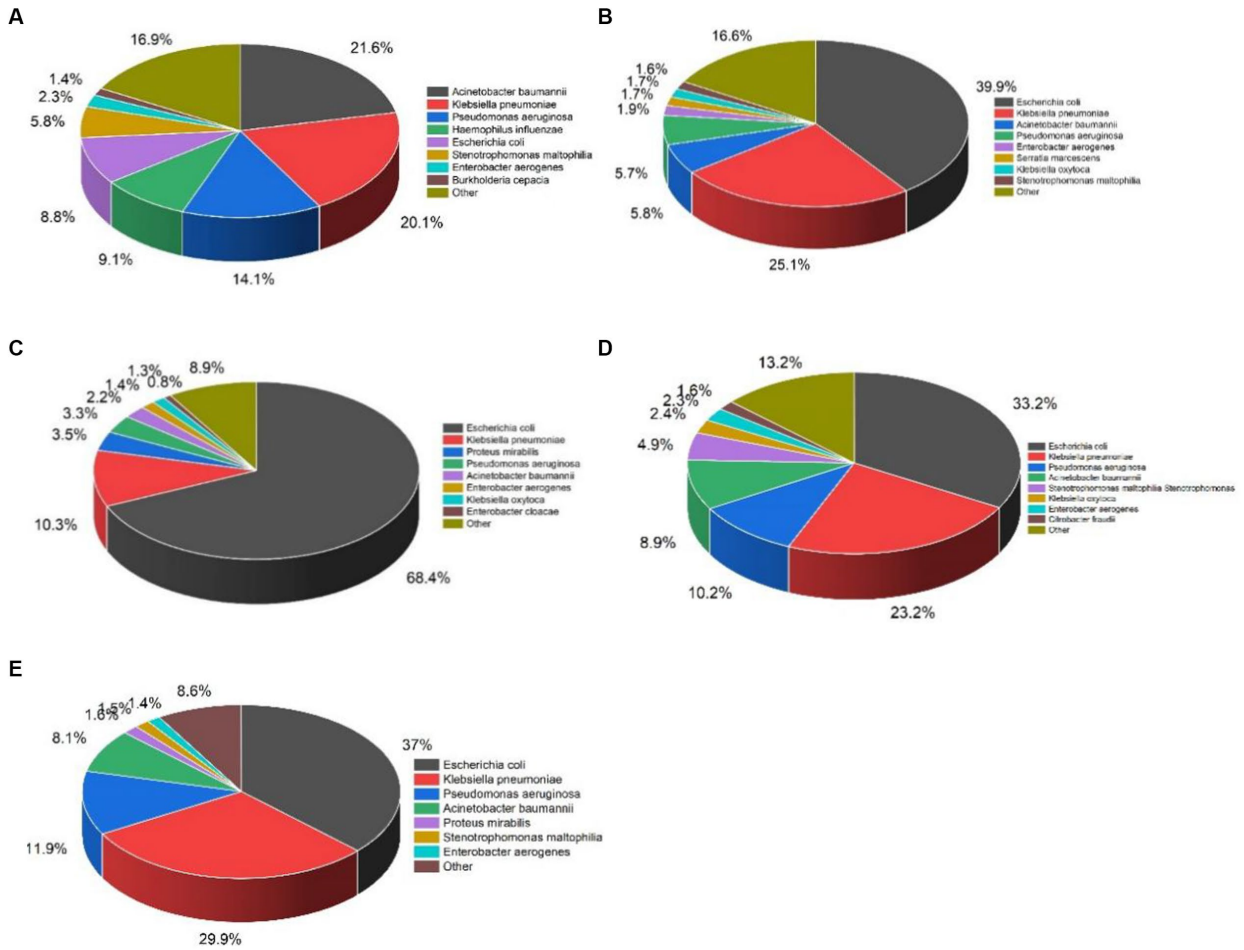
Distribution of organisms most frequently isolated from five specimen types between 2012 and 2021. *n* represents the total number of strains of gram-negative bacilli: **(A)** Respiratory tract, *n* = 22209; **(B)** Bloodstream, *n* = 3854; **(C)** Urinary tract, *n* = 8694; **(D)** Body fluid, *n* = 10307; **(E)** Wound and pus, *n* = 1182.

The distribution of organisms isolated in respiratory tract, bloodstream, urinary tract, body fluid, wound, and pus were shown in Figure 2. *Acinetobacter baumannii* (21.6%), *K. pneumoniae* (20.1%), and *P. aeruginosa* (14.1%) were the most common organisms isolated from respiratory tract, whereas *E. coli* accounted for only 8.8%. In contrast, *E. coli* was the top 1 organism isolated from urinary tract accounted for 68.4%, followed by *K. pneumoniae* (10.3%) and *P. mirabilis* (3.5%).The top three organisms isolated from bloodstream were *E. coli* (39.9%), *K. pneumoniae* (20.1%), and *A. baumannii* (5.8%). We noticed that *E. coli* was predominant in urine tract, bloodstream, body fluid, wound and pus, except for respiratory tract.

### Resistance profile of four major gram-negative bacilli

High resistance rate of *E. coli* to ampicillin, ciprofloxacin, levofloxacin, and trimethoprim sulfamethoxazole were observed, whereas amikacin, imipenem, and meropenem maintained remarkable activity to *E. coli* with the resistance rates fluctuated at 2%. Among all the antimicrobial agents tested, ampicillin had the highest and stable resistance rate, reaching more than 80%. The resistance rate to ciprofloxacin and levofloxacin significantly increased from 46.96 and 19.9% to 61.36 and 53.97%. In terms of cephalosporins, cefuroxime and ceftriaxone showed a downward trend with the high resistance rates dropped from 67.47 and 64.44% to 52.69 and 53.08%. Interestingly, both belonged to the third-generation cephalosporins, ceftazidime showed higher activity to *E. coli* than ceftriaxone. Besides the carbapenems and amikacin were the most active antimicrobial agents against *E. coli*, β-lactam/β-lactamase inhibitor combinations class was the third active antimicrobial agent. Piperacillin/tazobactam and cefoperazone/sulbactam with the resistance rates fluctuated at 10%, as shown in Table 2.

**Table 2:** Resistance rates (%) of *E. coli* to antimicrobial agents during 2012 to 2021.

Antimicrobial agent	2012 (*n* = 1,371)	2013 (*n* = 1,463)	2014 (*n* = 1,633)	2015 (*n* = 1,580)	2016 (*n* = 1,581)	2017 (*n* = 1,378)	2018 (*n* = 1,569)	2019 (*n* = 1715)	2020 (*n* = 1,588)	2021 (*n* = 1,611)
AMP	86.53	87.09	84.63	85.13	86.27	84.21	81.43	81.52	84.67	-
IPM	1.24	1.46	1.63	2.69	2.95	4.05	2.90	2.13	2.13	1.89
MEM	2.12	1.15	1.47	2.12	3.89	4.69	5.59	3.54	3.56	2.08
LVX	19.90	32.30	42.39	47.08	49.14	47.27	46.41	51.98	53.08	53.97
CIP	46.96	48.73	47.63	50.92	52.74	50.00	48.03	59.19	60.88	61.36
SXT	63.25	61.58	52.69	50.43	52.13	48.20	50.17	48.66	49.87	51.68
TZP	6.00	5.14	3.46	3.66	4.78	5.31	9.76	10.62	11.13	9.08
SCF	4.80	2.91	3.92	10.73	8.95	15.35	16.91	13.86	11.16	9.56
AMK	10.22	3.18	2.24	2.42	1.53	1.80	1.68	1.59	1.66	1.82
TOB	32.04	11.94	11.79	11.32	13.34	12.58	10.90	9.49	13.13	11.28
CN	44.53	33.00	39.22	37.87	39.57	34.29	28.78	27.96	29.02	-
ATM	56.74	48.03	57.68	44.22	45.62	43.63	40.00	42.34	41.49	41.99
CXM	67.47	65.15	57.00	50.00	55.45	62.96	61.24	59.23	56.69	52.69
CRO	64.44	67.10	60.32	62.88	63.68	60.94	56.88	57.19	55.19	53.08
CAZ	34.40	25.64	26.98	29.04	30.80	31.38	32.21	33.27	29.97	29.92
FEP	22.95	24.57	35.15	25.96	25.71	27.64	31.28	41.67	35.72	36.79
FOX	16.27	14.66	11.68	15.38	12.50	14.19	19.14	15.04	10.95	9.23

AMP, Ampicillin; IPM, Imipenem; MEM, Meropenem; LVX, Levofloxacin; CIP, Ciprofloxacin; SXT, Trimethoprim-sulfamethoxazole; TZP, Piperacillin/tazobactam; SCF, Cefoperazone/sulbactam; AMK, Amikacin; TOB, Tobramycin; CN, Gentamicin; ATM, Aztreonam; CXM, Cefuroxime; CRO, Ceftriaxone; CAZ, Ceftazidime; FEP, Cefepime; and FOX, Cefoxitin.

**Figure 3:**
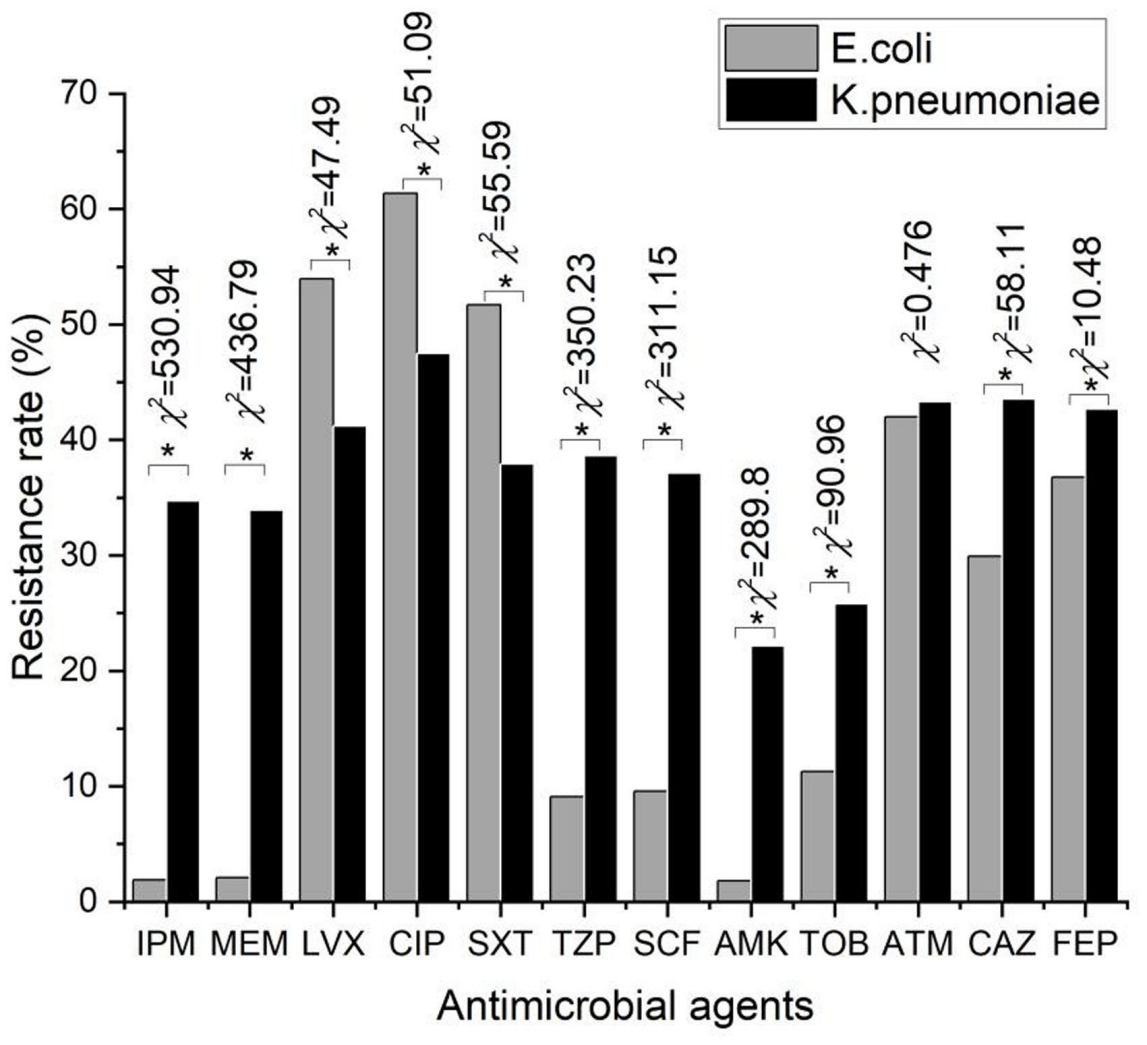
Comparison of 12 antibiotics resistance rates between *Escherichia coli* and *Klebsiella pneumoniae*. ^*^ represents the difference was statistically significant (*p* < 0.05).

As shown in Figure 3, the resistance rates of *E. coli* and *K. pneumoniae* to aztreonam were not significantly different. Moreover, excluding fluoroquinolones and trimethoprim-sulfamethoxazole, the resistance rates of *K. pneumoniae* to tested antimicrobial agents were generally higher than *E. coli* and the differences were statistically significant (*p* < 0.05). The dramatic increases of resistance were observed in carbapenems and fluoroquinolones over the past 10 years. In 2021, compared with *E. coli*, the resistance rates of *K. pneumoniae* to meropenem and imipenem were more than 15 times higher, reaching more than 30%.While the resistance rates of ciprofloxacin and levofloxacin were lower than *E. coli*. Additionally, resistance to piperacillin/tazobactam, cefoperazone/sulbactam, and amikacin also showed a gentle upward trend from 12.37, 7.66, and 8.76% in 2012 to 38.62, 37.11, and 22.17% in 2021. Fluctuations were found in trimethoprim-sulfamethoxazole, tobramycin, gentamicin, and aztreonam resistance rate to some extent but tended to be stable. The resistance rates of ceftazidime, ceftriaxone, and cefepime were more than 40% in 2021, as shown in Table 3.

**Table 3:** Resistance rates (%) of *K. pneumoniae* to antimicrobial agents during 2012 to 2021.

Antimicrobial agent	2012 (*n* = 689)	2013 (*n* = 663)	2014 (*n* = 818)	2015 (*n* = 689)	2016 (*n* = 731)	2017 (*n* = 861)	2018 (*n* = 1,054)	2019 (*n* = 1,494)	2020 (*n* = 1,512)	2021 (*n* = 1,586)
IMP	3.88	4.05	11.46	10.95	11.16	25.09	29.59	32.24	31.45	34.72
MEM	2.17	6.16	12.74	11.46	13.86	28.89	33.53	32.92	31.59	33.93
LVX	4.76	8.50	11.84	15.25	20.05	34.03	35.17	42.33	42.56	41.20
CIP	16.28	12.58	15.86	18.49	24.01	36.16	38.40	51.29	51.32	47.51
SXT	44.82	43.17	26.24	28.85	28.60	29.89	35.31	31.66	36.17	37.91
TZP	12.37	11.68	14.20	11.70	16.78	30.54	38.46	38.10	39.37	38.62
SCF	7.66	6.46	13.26	17.79	21.83	30.64	40.52	37.64	35.81	37.11
AMK	8.76	5.65	5.20	7.25	6.06	21.89	13.88	18.00	17.45	22.17
TOB	16.12	7.38	8.87	11.11	8.86	25.63	17.69	21.83	21.60	25.82
CN	23.32	18.58	18.88	20.71	20.51	30.86	-	-	-	-
ATM	48.52	38.46	43.62	45.28	49.30	50.46	50.72	48.79	46.40	43.31
CAZ	40.53	27.09	28.85	25.68	32.16	42.69	48.52	48.00	44.60	43.55
CRO	48.42	50.57	43.48	44.84	48.02	53.50	61.69	55.41	-	-
FEP	14.55	21.00	38.34	42.01	46.39	43.97	48.31	47.99	45.36	42.68

*Acinetobacter baumannii* was a gram-negative bacilli with the highest resistance rate to antimicrobial agents, and its resistance rate to all tested antimicrobial agents will reach more than 50% in 2020. The resistance rates of *A. baumannii* to imipenem and meropenem remarkably increased from 55.07 and 57.25% in 2012 to 78.67 and 79.62% in 2021. These increases were statistically significant (Imipenem: χ^2^ value was 64.86, *p* value was 0.000. Meropenem: χ^2^ value was 54.53, *p* value was 0.000). Fluoroquinolones, β-lactam/β-lactamase inhibitor combinations class, ceftazidime, and cefepime also had the same trend of change, the resistance rate has reached more than 70%. Moreover, the resistance rate of cefoperazone/sulbactam increased 53.3% over the 10 years whereas trimethoprim-sulfamethoxazole, amikacin, tobramycin, and gentamicin tended to be stable as shown in Table 4.

**Table 4:** Resistance rates (%) of *A. baumannii* to antimicrobial agents during 2012 to 2021.

Antimicrobial agent	2012 (*n* = 321)	2013 (*n* = 326)	2014 (*n* = 364)	2015 (*n* = 520)	2016 (*n* = 459)	2017 (*n* = 667)	2018 (*n* = 668)	2019 (*n* = 907)	2020 (*n* = 1,210)	2021 (*n* = 1,118)
IMP	55.07	64.53	58.55	59.08	61.51	72.92	70.70	72.39	77.80	78.67
MEM	57.52	67.70	60.50	70.43	73.35	75.90	74.40	73.65	78.68	79.62
LVX	42.43	48.91	40.53	39.48	46.83	52.49	61.65	65.85	72.02	73.69
CIP	56.19	65.02	56.23	58.08	61.51	71.41	70.97	66.36	77.77	78.41
SXT	58.91	60.86	47.40	42.28	46.90	63.14	54.47	53.25	56.68	48.69
TZP	59.23	68.37	57.51	55.20	55.95	79.43	74.22	73.11	79.11	79.86
SCF	16.78	13.06	16.22	36.29	28.96	62.27	62.31	65.44	72.56	70.08
AMK	58.53	66.22	41.67	45.25	48.73	41.81	55.56	50.11	53.29	-
TOB	60.87	57.38	50.14	45.51	52.38	68.01	59.04	61.72	56.58	65.59
CN	62.88	60.53	54.62	46.51	55.16	69.72	68.7	63.3	66.81	-
ATM	73.91	76.57	75.22	76.91	74.90	96.59	99.65	98.83	87.31	-
CAZ	60.20	66.09	60.68	54.64	57.14	67.32	71.40	73.25	78.61	78.92
CRO	75.51	66.24	65.03	61.77	64.80	72.06	50.00	55.29	64.29	-
FEP	55.43	67.33	61.85	59.28	63.49	71.45	63.79	66.50	74.37	73.84

As listed in Table 5, amikacin, tobramycin, and gentamicin showed high sensitivity to *P. aeruginosa*, and their respective resistance rates dropped from 14.48 to 5.48%, 18.38 to 6.54%, and 18.94 to 8.65%. Levofloxacin and ciprofloxacin had lower rates of resistance than the three gram-negative bacilli mentioned above, at 24.30 and 14.12% in 2021, respectively. While the resistance to carbapenem antibiotics like imipenem and meropenem was high overall, the resistance rate remained stable or showed a lower trend in the past 3 years. The resistance rate to ceftazidime and cefepime also demonstrated a negative trend.

**Table 5:** Resistance rates (%) of *P. aeruginosa* to antimicrobial agents during 2012 to 2021.

Antimicrobial agent	2012 (*n* = 383)	2013 (*n* = 440)	2014 (*n* = 519)	2015 (*n* = 527)	2016 (*n* = 486)	2017 (*n* = 496)	2018 (*n* = 559)	2019 (*n* = 701)	2020 (*n* = 788)	2021 (*n* = 744)
IMP	27.30	29.37	26.45	30.43	25.44	33.26	43.56	44.08	43.79	40.71
MEM	24.77	21.73	22.44	25.18	33.28	35.45	36.45	31.13	31.51	26.76
LVX	20.6	18.1	8.64	10.30	9.42	8.87	15.79	22.24	27.94	24.30
CIP	16.71	20.24	11.94	14.03	13.94	9.89	13.70	13.21	16.78	14.12
TZP	22.25	23.74	10.73	14.14	12.68	12.20	5.88	15.88	30.18	17.77
SCF	12.82	10.57	6.59	18.61	18.18	17.87	21.91	19.73	21.70	19.36
AMK	14.48	13.30	4.04	6.35	6.29	4.19	5.65	3.97	3.52	5.48
TOB	18.38	17.81	6.26	11.24	9.06	4.83	5.76	4.90	4.33	6.54
CN	18.94	20.00	8.89	12.85	10.14	5.58	6.67	8.57	10.34	8.65
CAZ	30.36	28.64	18.42	18.29	18.73	22.15	22.54	21.78	20.05	19.76
FEP	18.10	16.67	11.56	14.82	12.54	13.63	9.79	7.44	6.77	10.55

### Change of resistance of four major clinically isolated gram-negative bacilli to carbapenems

**Figure 4:**
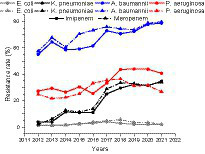
Change of resistance of four major clinically isolated gram-negative bacilli to imipenem and meropenem from 2012 to 2021.

As shown in Figure 4 and Tables 6, 7, of the four major gram-negative bacilli, *E. coli* had the lowest resistance rate to imipenem and meropenem, and the resistance rate remained stable over the past 10 years, fluctuating at 2%. The *p* value of linear correlation of *K. pneumoniae, A. baumannii*, and *P. aeruginosa* were less than 0.5, Pearson *R* > 0, indicating that the resistance rates to imipenem and meropenem have shown an increasing trend over the past 10 years. *Acinetobacter baumannii* had the highest resistance rate, reaching 78.67 and 79.62% in 2021. *Acinetobacter baumannii* and *K. pneumoniae* both showed rapid increases in carbapenem resistance, with *K. pneumoniae* showing an increase of more than 30%. It was important to note that *P. aeruginosa*’s resistance to imipenem and meropenem grew prior to 2018 and gradually declined following 2018.

**Table 6:** The resistance rates of four major GNBs to imipenem from 2012 to 2021.

Resistance rate of imipenem (%)	2012	2013	2014	2015	2016	2017	2018	2019	2020	2021	Pearson χ^2^	Pearson *R* value	*p* value
*E. coli*	1.24	1.46	1.63	2.69	2.95	4.05	2.9	2.13	2.13	1.89	25.182	/	0.647
*K. pneumoniae*	3.88	4.05	11.46	10.95	11.16	25.09	29.59	32.24	31.45	34.72	483.55	0.230	0.000
*A. baumannii*	55.07	64.53	58.55	59.08	61.51	72.92	70.7	72.39	77.8	78.67	167.78	0.159	0.000
*P. aeruginosa*	27.3	29.37	26.45	30.43	25.44	33.26	43.56	44.08	43.79	40.71	117.80	0.132	0.000

**Table 7:** The resistance rates of four major GNBs to meropenem from 2012 to 2021.

Resistance rate of meropenem (%)	2012	2013	2014	2015	2016	2017	2018	2019	2020	2021	Pearson χ^2^	Pearson *R* value	*p* value
*E. coli*	2.12	1.15	1.47	2.12	3.89	4.69	5.59	3.54	3.56	2.08	57.79	0.034	0.001
*K. pneumoniae*	2.17	6.16	12.74	11.46	13.86	28.89	33.53	32.92	31.59	33.93	426.05	0.219	0.000
*A. baumannii*	57.52	67.7	60.5	70.43	73.35	75.9	74.4	73.65	78.68	79.62	130.71	0.138	0.000
*P. aeruginosa*	24.77	21.73	22.44	25.18	33.28	35.45	36.45	31.13	31.51	26.76	56.32	0.060	0.000

### The rate of MDR and XDR in *Klebsiella pneumoniae*, *Acinetobacter baumannii*, and *Pseudomonas aeruginosa*

As shown in Table 8, *A. baumannii* exhibited the highest rate of MDR and XDR, reaching 60–80 and 45–55%, respectively. The rate of MDR in *K. pneumoniae* has also reached around 50%. Moreover, the rate of XDR in *K. pneumoniae* has increased during the study period, from 2.18% in 2012 to 21.67% in 2021. In comparison, the rate of MDR and XDR in *P. aeruginosa* have fluctuated to some extent, but overall they still tend to be stable.

**Table 8:** Rates (%) of MDR and XDR in *K. pneumoniae*, *A. baumannii*, and *P. aeruginosa.*

Type	*K. pneumoniae*		*A. baumannii*		*P. aeruginosa*	
	MDR	XDR	MDR	XDR	MDR	XDR
2012	59.65	2.18	64.55	46.15	36.77	8.64
2013	58.52	1.96	70.16	56.07	38.24	7.60
2014	48.79	8.51	63.58	51.16	28.51	5.02
2015	48.19	6.24	65.07	47.31	32.45	5.31
2016	52.56	6.98	64.29	50.79	21.25	7.32
2017	56.09	4.43	74.36	50.64	26.93	4.38
2018	54.43	4.18	72.67	50.33	30.26	5.83
2019	53.55	8.70	73.53	50.86	34.12	5.19
2020	58.83	17.23	78.97	54.15	35.18	4.45
2021	53.55	21.67	79.92	50.70	28.89	6.66

Resistance profiles of four major gram-negative bacilli isolated from ICU and non-ICU department.

The comparison of resistance rates of *E. coli*, *K. pneumoniae*, *A. baumannii*, and *P. aeruginosa* isolated from ICU and non-ICU department were showed in Table 9. Obviously, these four gram-negative bacilli isolated from ICU department exhibited significantly higher resistance rates than those from non-ICU department. These differences were statistically significant (*p* < 0.05). Among them, *K. pneumoniae* and *A. baumannii* were the most prominent, the resistance rate was 20–30% higher, while *E. coli* and *P. aeruginosa* were only 5–15% higher.

**Table 9:** Resistance rates (%) of four major gram-negative bacilli isolated from ICU and non-ICU departments.

Type	*E. coli*				*K. pneumoniae*				*A. baumannii*				*P. aeruginosa*			
	ICU	Non-ICU	χ^2^	*p*	ICU	Non-ICU	χ^2^	*p*	ICU	Non-ICU	χ^2^	*p*	ICU	Non-ICU	χ^2^	*p*
IPM	7.46	2.17	83.55	0.000	47.95	17.25	793.79	0.000	87.08	54.51	746.43	0.000	47.24	31.31	117.47	0.000
MEM	6.84	2.29	40.03	0.000	49.69	18.41	655.75	0.000	88.80	57.53	584.10	0.000	39.27	24.72	79.01	0.000
LVX	52.51	45.54	15.35	0.000	50.14	25.15	470.00	0.000	77.20	45.39	578.66	0.000	25.30	14.92	64.00	0.000
CIP	58.66	51.79	14.29	0.000	53.21	30.39	361.97	0.000	86.45	54.35	712.70	0.000	20.89	11.68	71.65	0.000
TZP	13.86	6.60	63.61	0.000	51.32	22.72	637.59	0.000	87.21	56.24	654.53	0.000	22.19	15.26	33.02	0.000
SCF	15.51	8.85	35.52	0.000	51.37	22.48	601.35	0.000	68.63	40.55	419.48	0.000	22.18	15.40	27.66	0.000
AMK	4.03	2.63	6.22	0.000	30.48	9.46	594.24	0.000	64.43	46.15	26.90	0.000	9.03	4.96	30.54	0.000
TOB	19.06	13.40	20.36	0.000	34.62	13.16	492.59	0.000	73.45	44.83	489.44	0.000	11.27	6.86	26.97	0.000
CN	43.28	36.59	10.29	0.001	29.60	20.62	25.08	0.000	76.14	45.07	210.53	0.000	16.40	11.19	13.56	0.000
CAZ	39.93	30.59	33.33	0.000	56.79	35.28	312.27	0.000	87.61	54.98	684.24	0.000	24.59	20.28	11.58	0.000
FEP	44.62	32.54	53.27	0.000	58.86	35.45	373.06	0.000	83.77	52.59	649.50	0.000	15.65	9.58	38.94	0.000
CRO	74.31	60.57	421	0.000	56.82	47.70	20.27	0.000	84.32	51.96	207.20	0.000	-	-		
ATM	57.86	45.80	43.52	0.000	60.44	41.92	218.67	0.000	95.03	81.59	112.98	0.000	-	-		
SXT	59.39	51.73	18.68	0.000	40.37	32.00	51.01	0.000	64.51	40.91	324.37	0.000	-	-		
CXM	73.82	63.50	8.30	0.002	-	-			-	-			-	-		
FOX	22.18	15.87	6.82	0.007	-	-			-	-			-	-		
AMP	91.09	84.77	16.6	0.000	-	-			-	-			-	-		

## Discussion

Since antimicrobial resistance has become a major challenge in the global public health field, mounting antimicrobial resistance among GNB causes empiric therapies troublesome since the limited number of antimicrobial agents are effective to control infections due to these resistant organisms. Given the fact that different countries and areas have their unique resistant pattern of organisms, surveillance of local resistant patterns will assist in guiding the judicious use of antimicrobial agents and in controlling antimicrobial resistance. In this paper, we conducted the surveillance study in central China during 2012–2021, while the distribution pattern and local antimicrobial-resistant trends of GNB over the past 10 years have been assessed.

The most frequently isolated GNB were *E. coli* (31.4%) and *Klebsiella* spp. (21.2%) followed by *Acinetobacter* spp. (13.8%), *P. aeruginosa* (11.0%). Moreover, *E. coli* was the top organism isolated from the urinary tract (68.4%), bloodstream (39.9%), body fluid (33.2%), and wound and pus (37%), except for the respiratory tract (8.8%). Whereas *A. baumannii* and *K. pneumoniae* were the major isolated organisms from the respiratory tract. It is suggested that *E. coli* mainly caused urinary tract infection, bloodstream infection, intra-abdominal infection, and wound infection, but less respiratory tract infection, which is consistent with the distribution of *E. coli* in the Asia-Pacific region (Lu et al., 2012), Latin-American (Gales et al., 2012), and Southern Africa (Brink et al., 2012). Notably, although *E. coli* was the highest isolation rate organism, the isolation rate showed a fluctuating downward trend in the past 10 years. Whereas the isolation rate of *K. pneumoniae* and *A. baumannii* displayed an overall upward trend except for a decline in 2021, which may be related to the severer antimicrobial resistance of *A. baumannii* and *K. pneumoniae* than that of *E. coli*.

Carbapenems were the most effective antimicrobial agents and were considered the last retort to defend against severe infections of GNB. With the development of carbapenemase, the global isolation rate of GNB resistant to carbapenems is growing. The class A carbapenemase KPC-2 was the most common type in China and was widely prevalent in *Enterobacteriaceae*, especially in carbapenem-resistant *K. pneumoniae* (Chen et al., 2011). The alarming findings observed in our study were *A. baumannii* and *K. pneumoniae* both proved rapid increases in carbapenem resistance, with the resistance rate of *K. pneumoniae* increasing from around 3% in 2012 to around 30% in 2021.The difference with our result was that *K. pneumoniae* had lower resistance rate in Germany, Japan, and Canada with 3.1, 1.8, and 1.3%, respectively. Whereas it had higher resistance rate in India, Greece, and Argentina, with 54.9, 53.6, and 46.6%, respectively (Lee et al., 2022). In 2021, the resistance rate of *A. baumannii* to carbapenems reached over 75%, lower than the resistance rate of over 95% in northeastern Iran (Mirzaei et al., 2020), and much higher than that of *A. baumannii* in the United States (Gupta et al., 2019), Switzerland (Ramette and Kronenberg, 2018), and Japan (Ueda et al., 2023), with lower resistance rates of 37.48, 8.9, and 0.9%, respectively. These low resistance rates was attributed to the effective strategies implement for controlling antibiotic use, such as changing the recommended, restricted, and off-supervised broad-spectrum antibiotic categories against gram-negative bacteria every 3 months based on the usage rate of these antibiotics. For carbapenem-resistant *A. baumannii*, long course of colistin therapy was proven to reduce the mortality rate of 30-day and have better clinical and microbiological outcomes (Katip et al., 2023). Fortunately, *E. coli* had the lowest resistance rate to imipenem and meropenem, and the resistance rate remained stable over the past 10 years, fluctuating at 2%, while this result is in line with other studies in Europe, Asia, and Latin America (Morrissey et al., 2013).The emergency of muti-drug resistant and extensively-drug resistant in *K. pneumoniae*, *A. baumannii*, and *P. aeruginosa* have become a public health threat. Our results found *A. baumannii* exhibited the highest rate of MDR and XDR, reaching 60–80 and 45–55%, respectively. Consistent with the west of Iran’s results that were 84 and 48% respectively (Hatami, 2018). Whereas the study published from United States (Mirzaei et al., 2020) and Japan (Ramette and Kronenberg, 2018) reported the rate of MDR in *A. baumannii* was 47.66 and 1.9%. *Klebsiella pneumoniae* also had high rate of MDR reached around 50%. Moreover, the rate of MDR in *P. aeruginosa* in our study (31.26%) was higher than that in United States (15.4%; Sader et al., 2017). The improper use of antibiotics in our hospital may be the cause of this difference.

In recent years, the incidence rate of infection caused by extended-spectrum β-lactamases-producing (ESBL) Enterobacteriaceae has increased worldwide, which can lead to nosocomial infections and even epidemic outbreaks. Under our surveillance, the resistance rate of *E. coli* and *K. pneumoniae* to ceftriaxone and ceftazidime was similar in Latin America (Gales et al., 2012). Interestingly, both belonged to the third-generation cephalosporins, ceftazidime demonostrated higher activity to *E. coli* and *K. pneumoniae* than ceftriaxone, which is because CTX-M was the most prevalent ESBL in China, which possesses stronger hydrolytic activity to ceftriaxone than ceftazidime (Pitout and Laupland, 2008). Moreover, a previous study reported the resistance plasmid that carries resistant genes encoding ESBL would meanwhile carry other drug-resistant genes, such as fluoroquinolones and aminoglycosides (Overdevest et al., 2011). Fluoroquinolones, particularly ciprofloxacin and levofloxacin were once used to be the first-line antimicrobial agents for the empirical treatment of complicated and uncomplicated urinary tract infection (UTI) and cystitis (Hooton, 2012). *Escherichia coli* and *K. pneumoniae* revealed substantial resistance rates and a growing tendency to ciprofloxacin and levofloxacin, which shows that fluoroquinolones are no longer the first-line treatment for *E. coli* infections. Conversely, amikacin showed high susceptibility to *E. coli* (97.2%), *K. pneumoniae* (87.4%), and *P. aeruginosa* (93.3%) except for *A. baumannii* (48.8%) which were significantly related to antimicrobial use. Hsueh et al. (2005) demonstrated that the decreasing use of amikacin was related to the increasing susceptibility of *P. aeruginosa* to amikacin, and the increased resistance to amikacin of *A. baumannii* was also associated with increased amikacin consumption. Remarkably, amikacin displayed comparable or superior activity with imipenem and meropenem, while these observations are following another study (Neuhauser et al., 2003). The combination of amikacin and β-lactam antimicrobial to treat severe infections caused by multi-drug resistant organisms had achieved a synergistic effect, reduced bacterial resistance and broadened the antibacterial spectrum (Ramirez and Tolmasky, 2017). Besides carbapenems and amikacin, the resistance rate of piperacillin/tazobactam and cefoperazone/sulbactam, cefoxitin were the third active antimicrobial to *E. coli* in our study.

The population residing in the ICU is vulnerable due to the presence of severe basic diseases, impaired host defenses, and diminished immunity (Brusselaers et al., 2011). Additionally, multiple surgeries and the use of invasive devices, such as mechanical ventilation, tracheal cannula, arterial catheter, central venous catheter, etc., can increase the risk of infection and colonization by MDR organisms (MacVane, 2017). Infections caused by MDR organisms have increased in the ICU, and it is difficult to select effective antimicrobial agents to treat them promptly, which is directly related to high morbidity, mortality, and hospitalization costs (Martin and Yost, 2011). In this study, we identified the troublesome situation. Four major GNBs isolated from the ICU department exhibited significantly higher resistance rates than those from those non-ICU departments, which was consistent with the United States and Europe (Sader et al., 2014). *Klebsiella pneumoniae* and *A. baumannii* were the most prominent, and the resistance rate was 20–30% higher while *E. coli* and *P. aeruginosa* were only 5–15% higher. The resistance rate of *A. baumannii* in the ICU to tested antimicrobial agents was the highest. Both belonging to non-fermenting bacilli, the resistance rates of *P. aeruginosa* were lower than *A. baumannii*. Aminoglycoside antimicrobials such as amikacin, tobramycin, and gentamicin proved the highest sensitivity to *P. aeruginosa*, with a downward trend of the resistance rate.

Detection of β-lactamases based on MIC and other phenotypic detection methods are imperfect (Livermore et al., 2012).When there is heterogeneous drug resistance or the expression of drug resistance phenotype is weak under *in vitro* experimental conditions, routine susceptibility testing is not precise. Furthermore, the results of routine susceptibility testing are relatively slow, delaying appropriate treatment and having adverse effects on clinical prognosis (Shorr et al., 2011; Katip et al., 2023). Therefore, the detection of drug resistance genes is particularly necessary. Drug resistance gene detection has the following advantages: Firstly, molecular genetics can directly start from samples without culturing of strain, which greatly saves time, provides guidance for clinicians to use antibiotics early and delays the development of microbial resistance. Secondly, it helps to identify results that are intermediate or have ambiguous results in routine susceptibility testing. Thirdly, in epidemiological tracking research of bacterial resistance, the detection of resistance genes is more accurate. Nevertheless, the detection of resistance genes faces enormous challenges. Currently, detection of resistance genes lacks standardized resistance gene databases and the testing cost is expensive. The final drug resistance phenotype is often caused by multiple drug resistance mechanisms, while molecular detection only relies on detecting one or several genes, resulting in inconsistent genotypes and phenotypes that cannot accurately guide anti-infection treatment. Therefore, it is of great significance to carry out accurate diagnosis and treatment of infectious diseases by combining drug resistance phenotype and drug resistance gene.

The abuse of antibiotics leads to increased selective pressure on bacteria, leading to bacterial mutations and the formation of drug resistance for survival. Antibiotics retain resistant bacteria and allow them to proliferate in large numbers. Once this resistance is acquired by other bacteria and passed on to the next generation, a large number of drug-resistant strains, even super bacteria, will be produced. Therefore, the rational use of antibiotics is particularly important for controlling bacterial resistance. At present, China’s antimicrobial stewardship policies mainly focus on second and third-level medical institutions, there is a lack of supervision and evaluation of grassroots communities, private hospitals, and private medical institutions. Therefore, it is recommended that the government formulate policies and guidelines for the rational use of antimicrobials in primary medical institutions.

There remain some limitations in our study. First, our study lacks an analysis of bacterial antimicrobial resistance genotypes and molecular typing. Second, the relationship between the consumption of antimicrobial agents and the resistance rate of antimicrobials has not been investigated. Despite these limitations, our surveillance study will not only help the hospital management department monitor the evolution of organisms’ resistance and strictly regulate the utilization rate and intensity of antimicrobials, but it will also assist clinicians in using antimicrobials sensibly in accordance with the local resistant pattern of organisms. Continuous surveillance of bacterial antimicrobial resistance trends is of utmost importance for the management of public health.

## Conclusion

*Escherichia coli*, *Klebsiella* spp., *Acinetobacter* spp., and *P. aeruginosa* were the most frequently isolated gram-negative bacilli. *Escherichia coli* was the top one organism isolated from urinary tract, bloodstream, body fluid, wound and pus, except for respiratory tract. Whereas *A. baumannii* and *K. pneumoniae* were the major isolated organisms from respiratory tract. *Acinetobacter baumannii* showed high resistance to the most commonly used antimicrobials. Ceftazidime showed higher activity to *Enterobacteriaceae* organisms than ceftriaxone. Amikacin, carbapenems and piperacillin/tazobactam exhibited relatively high sensitivity, whereas fluoroquinolones showed high resistance rate whether they can be the first-line antimicrobials for empirical treatment of UTI should take more consideration. *Acinetobacter baumannii* and *K. pneumoniae* showed rapid increases in carbapenem resistance whereas *E. coli* had the lowest resistance rate and remain stable at 2%. The gram-negative bacilli in ICU were more resistance than that in non-ICU. These findings are helpful for clinicians using antimicrobials reasonably.

## Data availability statement

The original contributions presented in the study are included in the article/supplementary material, further inquiries can be directed to the corresponding author.

## Ethics statement

The study was reviewed and approved by the institutional ethics board of Hunan Provincial People’s Hospital. All methods were carried out in accordance with relevant guidelines and regulations. Informed consent was obtained from all participants.

## Author contributions

TS: Data curation, Formal Analysis, Validation, Writing – original draft. LX: Data curation, Formal Analysis, Investigation, Supervision, Writing – review & editing.
